# The Effects of a Complex Interactive Multimodal Intervention on Personalized Stress Management Among Health Care Workers in China: Nonrandomized Controlled Study

**DOI:** 10.2196/45422

**Published:** 2024-07-12

**Authors:** Wenhua Liu, Quan Wang, Danli Zheng, Junhua Mei, Jiajia Lu, Guohua Chen, Wei Wang, Fengfei Ding

**Affiliations:** 1 Department of Pharmacology School of Basic Medical Science, Shanghai Medical College Fudan University Shanghai China; 2 Tongji Hospital Tongji Medical College Huazhong University of Science and Technology Wuhan China; 3 Department of Neurology, Wuhan No.1 Hospital Wuhan China

**Keywords:** multimodal intervention, stress management, health care workers, perceived stress, autonomic nervous system, stress, management, mental health, engagement, human support, physiological stress, psychological stress, social network, mobile phone

## Abstract

**Background:**

Health care workers (HCWs) frequently face multiple stressors at work, particularly those working night shifts. HCWs who have experienced distress may find it difficult to adopt stress management approaches, even if they are aware of the effects of stress and coping processes. Therefore, an individualized intervention may be required to assist distressed HCWs in bridging the “knowledge-practice” gap in stress management and effectively alleviating stress symptoms.

**Objective:**

The main objective of this research was to compare the effects of a complex interactive multimodal intervention (CIMI) to self-guided stress management interventions on stress symptoms of distressed HCWs, as measured by physiological (heart rate variability), psychological (perceived stress, mental distress, and subjective happiness), and sleep disorder (fatigue and sleepiness) indicators.

**Methods:**

We conducted a nonrandomized, controlled study in 2 Chinese general hospitals. The participants in this study were 245 HCWs who fulfilled at least 1 of the 3 dimensions on the Depression, Anxiety, and Stress Scale. All eligible individuals were required to complete a questionnaire and wear a 24-hour Holter device to determine the physiological signs of stress as indexed by heart rate variability at both baseline and after the intervention. The CIMI group received a 12-week online intervention with 4 components—mobile stress management instruction, a web-based WeChat social network, personalized feedback, and a nurse coach, whereas the control group simply received a self-guided intervention.

**Results:**

After a 12-week intervention, the Perceived Stress Scale (PSS) scores reduced significantly in the CIMI group (mean difference [MD] –5.31, 95% CI –6.26 to –4.37; *P*<.001) compared to the baseline levels. The changes in PSS scores before and after the intervention exhibited a significant difference between the CIMI and control groups (*d*=–0.64; MD –4.03, 95% CI –5.91 to –2.14; *P*<.001), and the effect was medium. In terms of physiological measures, both the control group (MD –9.56, 95% CI –16.9 to –2.2; *P*=.01) and the CIMI group (MD –8.45, 95% CI –12.68 to –4.22; *P*<.001) demonstrated a significant decrease in the standard deviation of normal-to-normal intervals (SDNN) within the normal clinical range; however, there were no significant differences between the 2 groups (*d*=0.03; MD 1.11, 95% CI –7.38 to 9.59; *P*=.80).

**Conclusions:**

The CIMI was an effective intervention for improving sleep disorders, as well as parts of the psychological stress measures in distressed HCWs. The findings provide objective evidence for developing a mobile stress management intervention that is adaptable and accessible to distressed HCWs, but its long-term effects should be investigated in future research.

**Trial Registration:**

ClinicalTrials.gov NCT05239065; https://clinicaltrials.gov/ct2/show/NCT05239065

## Introduction

### Overview

As a result of shift schedules, night shift health care employees face multiple workplace stressors, including insufficient rest periods, an increased risk of sleep deprivation, and mood changes [1-3]. Work-related stress has been shown to have a negative impact on employees’ physical health, including a greater likelihood of developing hypertension, the advancement of cardiovascular diseases, and all-cause and coronary heart disease mortality as measured by heart rate variability (HRV) [4-7]. The link between work-related stress and psychological distress is also well established [8], and according to a recent meta-analysis, the prevalence of depression, anxiety, and stress symptoms among health care workers (HCWs) was 24.3%, 25.8%, and 45%, respectively, which was higher than in other occupational populations [9]. Furthermore, HCWs who are under stress at work are more likely to experience sleep problems such as fatigue and sleepiness [10], which can jeopardize their job performance and patient safety.

During the past decade, numerous workplace-based stress management programs have been conducted in high-income countries and achieved the desired effect in improving the mental health and stress symptoms of staff [11,12]. However, stress management interventions remained scarce in the context of low- and middle-income countries [11]. On the one hand, due to the frequent conflict between increased medical service demand and limited health care resources, HCWs in these countries experienced a higher level of work-related stress than their counterparts [13]. On the other hand, limited medical resources made it difficult for distressed HCWs to seek professional help, and the current treatment gap for mental disorders in low- and middle-income countries is estimated to be from 72% to 93%, with those from the poorest countries receiving less coverage [14]. Therefore, it is imperative to take effective measures to assist distressed HCWs in these countries in reducing their work-related stress so that they do not endure.

For the time being, several stress management techniques, such as regular physical activity, deep breathing, and mindfulness, have been recommended for occupational populations at the workplace [15-17]. Building new stress management habits, however, may be difficult for HCWs, especially those with distress symptoms. Based on the findings of a recent mixed methods study, while the majority of HCWs were aware of some of the stress effects and stress management methods, almost half of them failed to adopt stress management behaviors [18]. Individuals enduring distress symptoms such as anxiety and depression may experience loneliness, isolation, and stigma, which can lead to stress-coping failure and increased stress responses [19]. Besides, the long-term distress symptoms may diminish their self-efficacy and perceived social support and hinder them from taking advantage of stress management [20]. It appears that a “stress-distress” vicious circle exists, preventing HCWs from putting stress management knowledge into practice [21]. As a result, it is critical to assist distressed HCWs in closing the “knowing-doing” gap and benefiting from stress management.

Knowledge translation is defined as a process that occurs through social and environmental interactions and underlines that knowledge exchange must happen in an interacting social situation [22]. Among different theoretical frameworks that are applied in knowledge translation research, the most popular makes reference to the social learning theory (SLT) [23]. The SLT emphasizes 3 main elements associated with human behaviors—environmental factors, personal factors, and behavioral factors [24], and only when environmental and individual factors interact synergically toward maintainability can knowledge translate to the desired behavioral changes [25-27]. In light of the theoretical foundation outlined, creating an online intervention context may help HCWs reap the most benefit from stress management and alleviate stress symptoms [28].

The internet can be considered ideal for creating an online context in stress management intervention. First, by introducing social learning via the internet, stressed individuals may be able to change their attitudes toward the treatment, such as feeling alone, isolated, or stigmatized [29]. Second, online peer interaction can provide a supporting environment where participants may find and provide practical advice, as well as receive social connections that may be lacking in their daily lives [30]. Third, the use of a mobile health device would enable participants to self-monitor and receive feedback on their performance and tension reduction, which could be beneficial for enhancing their stress management skills [31]. Fourth, human support and guidance provided by coaching may potentially alter individuals’ self-efficacy belief in stress management [32,33]. Besides, the high percentage of internet users suggests that it has potential applications in working populations—according to the most recent official report, China’s internet penetration rate has reached 73%, and there are more than 1.32 billion netizens in China, 99.7% of whom access the internet via smartphones [34]. Therefore, mobile stress management intervention may be an adaptable and accessible approach for distressed HCWs.

In response to the aforementioned findings, a complex interactive multimodal intervention (CIMI) for distressed HCWs was developed. The CIMI consisted of four components: (1) mobile stress management education, (2) a web-based social network provided by a WeChat (Tencent Holdings Limited) group, (3) individualized practice feedback, and (4) personalized support and guidance provided by a nurse coach.

### Objectives

Our primary objective was to figure out whether the CIMI is more effective than self-guided stress management in improving the stress symptoms of distressed HCWs, as measured by physiological (HRV indicators), psychological (perceived stress, mental distress, and subjective happiness), and sleep disorder (fatigue and sleepiness) indicators.

## Methods

### Study Design

The trial was a cluster-based, nonrandomized, parallel controlled study conducted in 2 hospitals in Wuhan, China, from August 11, 2021, to January 31, 2022. In order to prevent data contamination, we used a nonrandomized study design in this study. Hospital A participants received the CIMI intervention, while hospital B participants received the self-guided stress management intervention.

### Recruitment and Participants

All participants were recruited between August 11, 2021, and October 31, 2021. HCWs were eligible to participate if they (1) worked the night shift in hospital A or hospital B; (2) did not plan to leave their current position within the next 6 months; (3) did not have any serious clinical diagnoses that affected stress levels or follow-up quality (ie, abnormal cardiac rhythm; heart disease including coronary artery disease, angina, and arrhythmia; cardiac pacemaker; stroke; panic attack; and cognitive impairment); and (4) had at least 1 sign of psychological distress measured by Depression, Anxiety, and Stress Scale (DASS-21; ie, the subscale of depression, anxiety, and stress was above 10 points, 7 points, and 11 points, respectively) [35]. HCWs are excluded if they (1) were unwilling to complete the 3-month follow-up or (2) were unable to do exercise because of fractures or limb abnormalities, and so forth.

**Figure 1:**
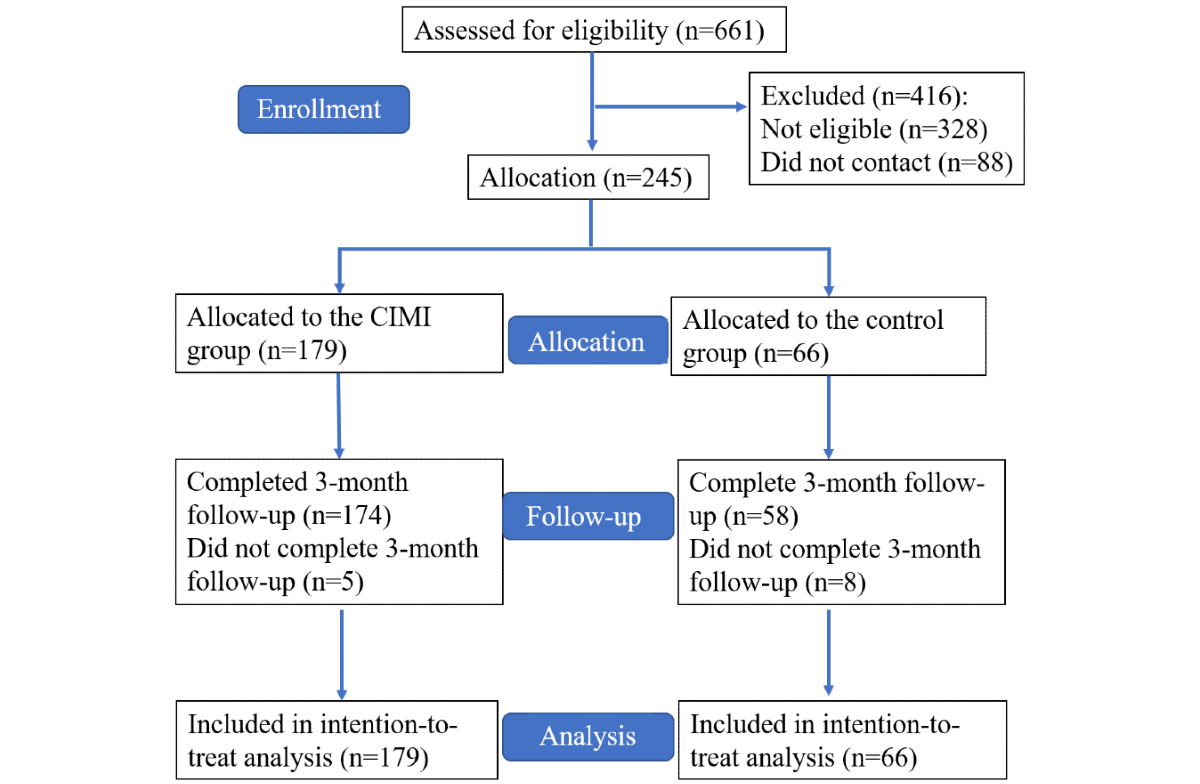
Participant flowchart. CIMI: complex interactive multimodal intervention.

Although we intended to recruit participants with a good sex and age balance, the COVID-19 pandemic caused numerous difficulties during the recruiting process. As a result, this study was unable to maintain a balanced age and sex distribution. In total, 661 participants completed the prescreening questionnaires, and 245 of them were eligible for enrollment based on the inclusion and exclusion criteria. Participants from hospital A (n=179) were assigned to the intervention group and participants from hospital B (n=66) were assigned to the control group. A total of 5 participants from the intervention group and 8 from the control group dropped out of the study during the 3-month study period due to personal reasons (Figure 1). According to Table 1, of the 245 participants, 208 (84.9%) were female, with a mean age of 33.7 (SD 0.3) years. In regard to educational background, 57 (23.3%) participants obtained graduate degrees. There were 85 (23.7%) doctors or other health care professionals and 160 (65.3%) nurses in terms of position. With respect to age, marital status, education, weekly working hours, and health status, the CIMI and control groups are comparable. In comparison to the control group, the CIMI group had a higher proportion of female participants (157/179, 87.7% vs 51/66, 77.3%; *P*=.04), those who worked for more than 10 years (98/179, 54.8% vs 23/66, 34.9%; *P*=.006), and nurses (122/179, 68.2% vs 38/66, 57.6%; *P*=.003).

**Table 1:** Baseline characteristics of the participants.

Variables		Total (N=245)	Intervention (n=179)	Control (n=66)	*P* value	
Age (years)^a^, mean (SD)		33.7 (0.3)	33.6 (0.4)	33.9 (0.7)	.67	
**Sex^b^, n (%)**						.04
	Female	208 (84.9)	157 (87.7)	51 (77.3)		
	Male	37 (15.1)	22 (12.3)	15 (22.7)		
**Marital status^b^, n (%)**						.79
	Unmarried	70 (28.6)	52 (29.1)	18 (27.3)		
	Married	175 (71.4)	127 (71)	48 (72.7)		
**Educational level^b^, n (%)**						.054
	≤Undergraduate	188 (76.7)	143 (79.9)	45 (68.2)		
	≥Postgraduate	57 (23.3)	36 (20.1)	21 (31.8)		
**Working experience (years)^b^, n (%)**						.006
	<10	124 (50.6)	81 (45.3)	43 (65.2)		
	≥10	121 (49.4)	98 (54.8)	23 (34.9)		
**Role^b^, n (%)**						.003
	Nurse	160 (65.3)	122 (68.2)	38 (57.6)		
	Physician	58 (23.7)	33 (18.4)	25 (37.9)		
	Other health care workers	27 (11)	24 (13.4)	3 (4.6)		
**Weekly working hours^b^ (hours), n (%)**						.76
	≤40	126 (51.4)	91 (50.8)	35 (53)		
	>40	119 (48.6)	88 (49.2)	31 (47)		
**Health status^b^, n (%)**						.47
	Bad	54 (22)	38 (21.2)	16 (24.2)		
	So-so	167 (68.2)	121 (67.6)	46 (69.7)		
	Good	24 (9.8)	20 (11.2)	4 (6.1)		
**Diagnosed disease^b^, n (%)**						.99
	No	193 (78.8)	141 (78.8)	52 (78.8)		
	Yes	52 (21.2)	38 (21.2)	14 (21.2)		

^a^Continuous variables.

^b^Categorical variables.

### Power Calculation

The primary outcomes in our study were Perceived Stress Scale (PSS) scores and HRV measurements. As reported in a previous study [36], the PSS score decreased by 1.5 points after intervention in the control group. We hypothesized that the PSS score in the CIMI group would decrease by an additional 2 points compared to the control group. Therefore, at least 60 participants per group were required to detect a difference between the 2 groups with 85% power, accounting for a 20% dropout rate and a significance level of 0.025.

Similarly, based on HRV indicators, the SD of all normal-to-normal intervals (SDNN) of the HRV indicator decreased by 5 ms^2^ in the control group after intervention [37]. We assumed that the CIMI group would experience a greater reduction in SDNN by 2 ms^2^ compared to the control group. Therefore, a minimum of 200 participants per group were required to detect a difference between the 2 groups with 85% power, accounting for a 20% dropout rate and a significance level of 0.025. Therefore, we planned to recruit 200 participants in each group to ensure the detection of differences in both PSS and SDNN between the 2 groups.

However, the recruitment process was impeded due to the COVID-19 pressures, and we eventually enrolled 245 people who satisfied our inclusion criteria. Hence, the power calculation in this study was determined via a post hoc analysis. According to our study, PSS scores fell by 1.28 points in the control group and 5.31 points in the CIMI group between the baseline and 3 months after the intervention, respectively. Therefore, taking into account a 2-sided significance level of .05, the sample sizes of 179 in the intervention group and 66 in the control group would have more than 95% power to detect the significant difference between the 2 groups.

### Procedure

First, a screening questionnaire about demographic characteristics (age, sex, marital status, educational level, working experience, role, and working hours), health status, diagnosed disease, and the DASS-21 was sent to the potential participants. After the screening, all eligible participants were required to complete a detailed baseline questionnaire and wear a 24-hour Holter device to assess the physiological indicators of stress as indexed by HRV. The day of HRV recording was selected as at least 48 hours after a night shift to ensure enough rest prior to recording. Furthermore, a sport wristband and corresponding use method were given to each participant, and participants were also asked to upload their exercise data from their wristbands to a research app on a daily basis. The research app only acted as a recording application that tracked daily information related to exercise, such as daily exercise time, consumed calories, and step counts, instead of providing any interaction between participants and researchers. In addition, a printed brochure was distributed to both the CIMI and control groups to teach them how to use 3 types of stress management techniques (ie, regular physical activity, deep breathing, and mindfulness; Multimedia Appendix 1).

During the 12-week intervention, the CIMI group was required to complete a weekly online questionnaire that contained 4 sections—daily life stressors, stress management practices, psychological symptoms, and sleep quantity and quality (Multimedia Appendix 2). The questionnaire was sent to each participant’s mailbox, and the nurse coach was responsible for checking and ensuring the completion of the questionnaire, as well as reminding participants if they were unable to complete it within 3 days of receiving it. To keep contact between researchers and participants to a minimum, the control group only needed to complete the online questionnaire biweekly. All questionnaire data were collected using the REDCap (Research Electronic Data Capture; Vanderbilt University) platform.

### Intervention

Participants in the CIMI group received a 12-week online intervention with the following 4 components: mobile stress management education, a web-based WeChat social network, tailored feedback, and a nurse coach.

### Mobile Stress Management Education

Participants in the CIMI group were invited to join the WeChat group anonymously. A professional nurse coach delivered the evidence-based course about stress through a WeChat group every weekend for 12 weeks. In addition, 3 evidence-based stress management strategies (SMSs)—physical activity, deep breathing, and mindfulness—were introduced during every course, and to ensure participants practiced those SMSs appropriately, each participant received a package of instructional videos to guide them through the practices’ techniques.

### Web-Based WeChat Social Network

During each course through the WeChat group, a 30-minute online discussion session was scheduled. During the discussion, participants were invited to answer several questions about stress management and encouraged to share their photos or videos that recorded their SMS practice over the past week.

### Tailored Feedback

The weekly and monthly summary reports on the stress symptoms and SMS performance were sent to the participants through email. The summary reports were developed by the research group based on the uploaded research app data from the wristbands and the weekly questionnaires completed by the participants.

### Nurse Coach

The nurse coach would help participants whenever needed to (1) ensure their proper use of portable devices and continuous participation in the program, (2) evaluate their practice performance and identify possible barriers to practicing SMSs and further give specific instructions, and (3) support HCWs and encourage them to practice SMSs continuously during the intervention.

### Control Group

The control group received the same wristband and research app, as well as instructions to ensure that they used the wristbands correctly. Participants in the control group were asked to fill out a brief online questionnaire every 2 weeks and upload their exercise data from the wristbands daily, but they would not receive the 4 components of intervention mentioned.

### Outcomes

#### Primary Outcomes

##### Perceived Stress

The PSS-14 is a globally used 14-item scale that has been translated and validated in the Chinese population to measure the level of perceived stress [38]. The PSS-14 used a 5-point Likert scale, and each item was scored from 0 to 4. The total score ranges from 0 to 56, with higher total scores indicating a higher level of stress [39]. The Cronbach α was 0.835, demonstrating that the PSS-14 was a reliable instrument to measure the participants’ perceived stress in this study.

##### HRV Metric

HRV is a common tool for assessing autonomic nervous system function, and it reflects the balance of the cardiovascular system controlled by the sympathetic and parasympathetic nerves of the autonomic nervous system [40]. The research and clinical device (DMS300-4, Diagnostic Monitoring Software) had an internal sampling rate of 4096 Hz, and the R-peaks were automatically detected with a precision of <1 ms. In this study, the SDNN, which is the most frequently used time domain measure of HRV, was used to assess physiological measures of stress [41,42]. The power spectrum of HRV was calculated by fast Fourier transform and expressed by ms^2^ power spectrum density [41]. To avoid signal quality issues from biological (ectopic beats, arrhythmia, etc) or technological (movement, signal interference, etc) sources, all electrocardiograph files were manually edited for detection by trained biomedical analysts. Values with differences greater than 20% were considered artifacts and the interpolation of degree zero was used for the artifact.

#### Secondary Outcomes

##### Mental Distress

The DASS-21 is a well-developed instrument used in the Chinese population for assessing the negative emotions of individuals in the past week [43]. The 21-item scale can be clustered into 3 subscales, and each subscale is rated on a 4-point Likert scale. The sum score of each subscale is multiplied by 2 and ranges from 0 to 42, with a higher score representing greater emotional health. The Cronbach α was 0.877, 0.872, and 0.805 for the depression subscale, anxiety subscale, and stress subscale, respectively, indicating excellent reliability for this scale in measuring emotional health in this study.

##### Subjective Happiness

The Subjective Happiness Scale (SHS) was used to assess the global subjective happiness of participants. The SHS consists of 4 items, and each item is rated on a 7-point Likert scale ranging from 1 to 7. Responses on this scale were summed and divided by 4, with higher scores indicating more happiness. The SHS has been used and validated in Chinese adults [44]. The Cronbach α was 0.683 for the SHS, demonstrating the reliability of this scale is acceptable in this study.

##### Fatigue Symptoms

The Fatigue Assessment Scale (FAS) is a widely used fatigue measure that includes 10 items rated on a 5-point Likert scale. This scale has been tested extensively on HCWs and demonstrated to have reliability and validity for measuring fatigue and to be free of gender bias [45]. The Cronbach α for the FAS was 0.883, indicating that the internal reliability of this scale was quite satisfactory in this study.

##### Sleepiness

The Epworth Sleepiness Scale (ESS) is a useful tool for evaluating daytime sleepiness in both the general population and shift workers [46]. This scale consists of 8 items, and each item is rated on a 4-point Likert scale. The Cronbach α for the ESS was 0.768, confirming that this scale is reliable for measure sleepiness in this study.

### Statistical Analysis

The analysis was conducted according to the intention-to-treat principle. Continuous variables were described by mean (SD) or median (IQR), and categorical variables were represented by frequency (percentage). Baseline characteristics were compared between groups using the 2-sample *t* test for normally distributed continuous variables, nonparametric Wilcoxon rank sum tests for nonnormally distributed variables, and the chi-square test for categorical variables. A linear mixed-effects regression model with random intercepts was fitted for each primary and secondary outcome. A 2-way interaction between the groups (control vs intervention) and time (baseline vs postintervention) was modeled using restricted maximum likelihood with the Kenward-Roger denominator degrees of freedom. As proposed by the Journal of Biomedical Informatics (JBI) checklist for quasi-experimental studies (nonrandomized experimental studies), the disparities between participants in compared groups pose a risk to the internal validity of a study investigating causal linkages. Furthermore, as indicated by 2 publications published in *the Journal of Medical Internet Research*, the authors also emphasized the significance of managing confounding variables to guarantee the comparability of the intervention and control groups. Therefore, in our statistical analysis approach, we included the age, sex, marital status, education level, working experience, occupation, weekly working hours, health status, and diagnosed disease as covariates in each model, to guarantee that the impact size can be fully attributed to our intervention and not influenced by other factors.

A post hoc exploratory subgroup analysis was performed to detect the influence of age, sex, marital status, education level, working experience, occupation, and weekly working hours by adding the 3-way interaction including group, time point, and subgroup variable into the model, respectively. The adjusted mean difference (MD) with 95% CI from baseline to after the intervention for the CIMI group and the control group separately, and the between-group adjusted MD with 95% CI from baseline to after the intervention were obtained from the linear mixed-effects regression. We used Cohen *d* as the standardized effect size, which is considered small (0.2), medium (0.5), or large (0.8), and the Cohen *d* was calculated with the between-group adjusted MD divided by the SDs at baseline. The missing values of the outcomes were handled in the mixed-effects models. All analyses were performed by the Stata 15.1 software (StataCorp LLC) at a 2-sided α level of .05.

### Ethical Considerations

The study was approved by the institutional review board of Tongji Medical College, Huazhong University of Science and Technology (IRB#2021S141). Before participating in the study, all participants provided informed consent in writing. All study data were deidentified to ensure participants’ privacy and confidentiality. A complementary cardiovascular assessment for a close relative was given to research participants who completed the program, while no monetary rewards were offered.

## Results

### Primary Outcome

As shown in Table 2, the PSS scores reduced significantly in the CIMI group (MD –5.31, 95% CI –6.26 to –4.37; *P*<.001) after the intervention as compared to the baseline levels, while the PSS scores of the control group remained largely unaltered (MD –1.28, 95% CI –2.91 to 0.34; *P*=.12). The changes in PSS scores before and after the intervention exhibited a significant difference between the CIMI and control groups (d=–0.64; MD –4.03, 95% CI –5.91 to –2.14; *P*<.001), and the effect was medium.

In terms of physiological measures, both the control group (MD –9.56, 95% CI –16.9 to –2.2; *P*=.01) and the CIMI group (MD –8.45, 95% CI –12.68 to –4.22; *P*<.001) demonstrated a significant decrease in SDNN within the normal clinical range (102-180 ms). In terms of the changes, there were no significant differences between the 2 groups (d=0.03; MD 1.11, 95% CI –7.38 to 9.59; *P*=.80).

**Table 2:** Estimations of adjusted mean differences and effect sizes for stress indicators (linear mixed model).

			Control group									Intervention group									Between group				
			Baseline, mean (SD)		Post intervention, mean (SD)		Change from baseline, adjusted MD^a^ (95% CI)^b^		*P* value		Baseline, mean (SD)			Post intervention, mean (SD)		Change from baseline, adjusted MD (95% CI)^b^		*P* value		Change from baseline, adjusted MD (95% CI)^c^			*P* value		Cohen *d* (95% CI)
**Physiological indicators**																									
	SDNN^d,e^	156.7 (35.2)		145.7 (34.6)		–9.56 (–16.9 to –2.22)		.01		156.8 (32.5)			147 (32.6)		–8.45 (–12.68 to –4.22)		<.001		1.11 (–7.38 to 9.59)			.80		0.03 (–0.25 to 0.32)	
**Psychological indicators**																									
	PSS^f^	30.7 (7.0)		29.2 (5.5)		–1.28 (–2.91 to 0.34)		.12		31.6 (6.3)			26.4 (6.3)		–5.31 (–6.26 to –4.37)		<.001		–4.03 (–5.91 to –2.14)			<.001		–0.64 (–0.93 to –0.36)	
	DASS-D^g^	11.4 (5.7)		10.3 (6.2)		–0.97 (–2.74 to 0.79)		.28		13.3 (8.0)			8.8 (6.8)		–4.5 (–5.52 to –3.47)		<.001		–3.53 (–5.57 to –1.48)			.001		–0.48 (–0.77 to –0.19)	
	DASS-A^h^	10.9 (5.6)		14.6 (6.6)		3.78 (1.97 to 5.59)		<.001		13.2 (6.3)			13.4 (6.8)		0.21 (–0.85 to 1.26)		.70		–3.57 (–5.67 to –1.47)			.001		–0.60 (–0.88 to –0.31)	
	DASS-S^i^	16.3 (6.4)		10.2 (6.3)		–6.09 (–8.0 to –4.17)		<.001		19.6 (6.8)			9.3 (5.9)		–10.33 (–11.45 to –9.22)		<.001		–4.25 (–6.46 to –2.03)			<.001		–0.64 (–0.93 to –0.36)	
	SHS^j^	3.7 (0.7)		4 (0.8)		0.26 (0.05 to 0.47)		.02		3.7 (0.9)			4.3 (1)		0.64 (0.52 to 0.76)		<.001		0.38 (0.14 to 0.63)			.002		0.47 (0.18 to 0.75)	
**Sleep indicators**																									
	ESS^k^	7.6 (3.2)		7.9 (4.3)		0.5 (–0.43 to 1.42)		.29		8.7 (3.9)			7.8 (4)		–0.81(–1.35 to –0.28)		.003		–1.31 (–2.39 to –0.24)			.02		–0.35 (–0.64 to –0.07)	
	FAS^l^	29.3 (4.8)		27 (4.8)		–2.04 (–3.42 to –0.67)		.004		30.8 (5.6)			26.3 (6.3)		–4.52(–5.32 to –3.72)		<.001		–2.48(–4.07 to –0.89)			.002		–0.47 (–0.75 to –0.18)	

^a^MD: mean difference.

^b^Change from baseline to after the intervention for intervention group and control group, respectively.

^c^Mean difference of change from baseline to after the intervention between intervention group and control group.

^d^SDNN: SD of normal-to-normal interval.

^e^The normal clinical range of SDNN was from 102 to 180 ms.

^f^PSS: Perceived Stress Scale.

^g^DASS-D: The Depression, Anxiety, and Stress-21 Scale’s Depression subscale.

^h^DASS-A: The Depression, Anxiety, and Stress-21 Scale’s Anxiety subscale.

^i^DASS-S: The Depression, Anxiety, and Stress-21 Scale’s Stress subscale.

^j^SHS: Subjective Happiness Scale.

^k^ESS: Epworth Sleepiness Scale.

^l^FAS: Fatigue Assessment Scale.

### Secondary Outcome

From baseline to after the intervention, the depression score of the CIMI group decreased by 3.53 points more than that of the control group (95% CI –5.57 to –1.48; *P*=.001; d=–0.48), and the stress score decreased by 4.25 points more than that of the control group (95% CI –6.46 to –2.03; *P*<.001; d=–0.64). Nevertheless, the anxiety score increased significantly in the control group (95% CI 1.97-5.59; *P*<.001), whereas that did not change significantly in the CIMI group (95% CI –0.85 to 1.26; *P*=.70). As compared with the control group, the CIMI group had statistically significant decreases in fatigue symptoms (MD –2.48, 95% CI –4.07 to –0.89; *P*=.002; d=–0.47) and daytime sleepiness (MD –1.31, 95% CI –2.39 to –0.24; *P*=.02; d=–0.35), in addition to a significant increase in subjective happiness (MD 0.38, 95% CI 0.14-0.63; *P*=.002; *d*=0.47), before and after the intervention (Table 2). Overall, the CIMI group had a medium effect on improving depression, stress, fatigue, and promoting subjective happiness, a small effect on improving sleepiness.

### Subgroup Analysis

**Figure 2:**
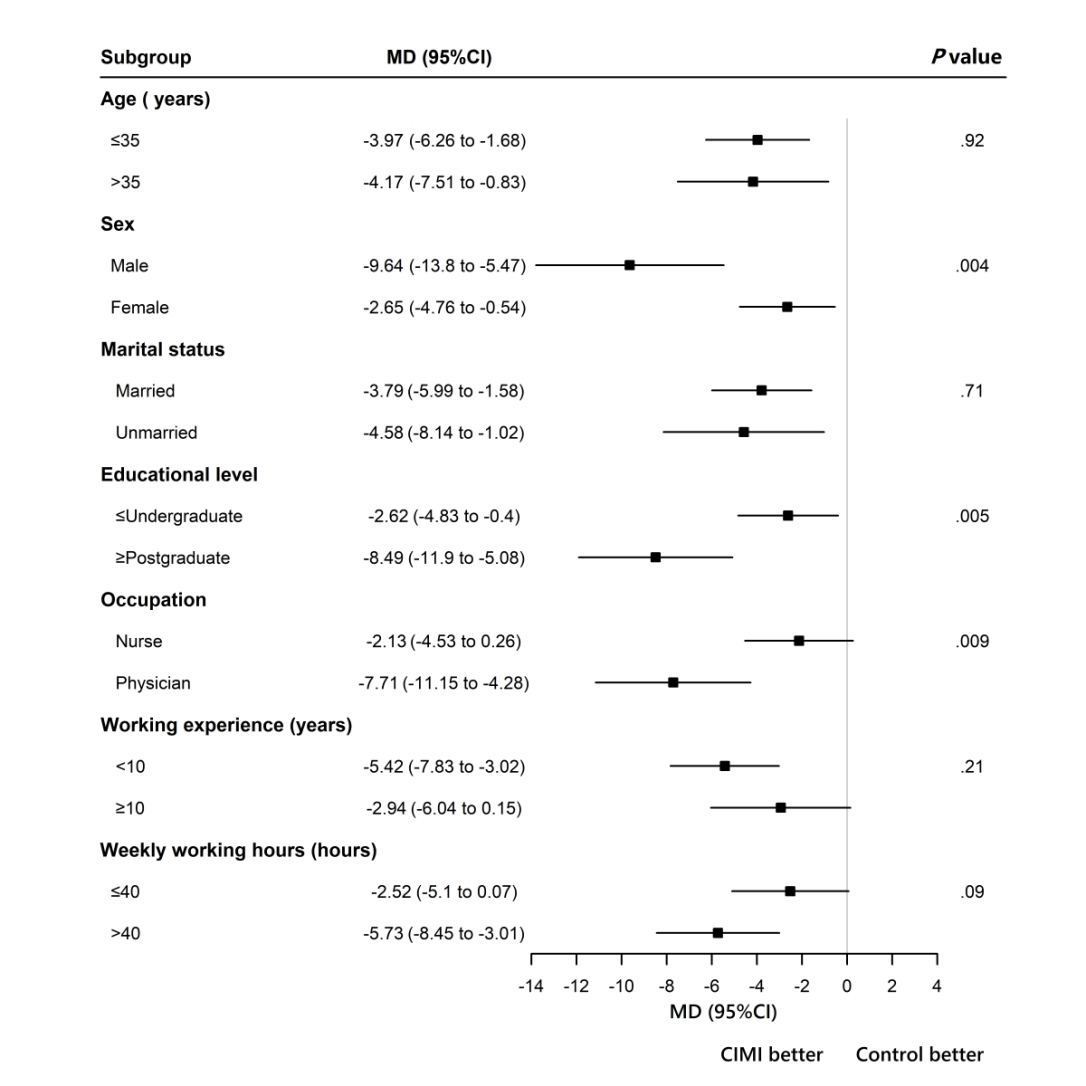
Subgroup analysis to detect the influence of demographic factors on treatment effects. CIMI: Complex Interactive Multimodal Intervention; MD: mean difference.

Subgroup analyses of the PSS score were conducted based on the age, sex, marital status, education levels, working experience, occupation, and weekly working hours of the participants. As shown in Figure 2, the effect sizes between the CIMI group and the control group were statistically better in male than female individuals, better in doctors than nurses, and higher in participants with postgraduate degrees than those with bachelor’s degrees (interaction *P* values for sex=.004, *P* values for occupation=.005, and *P* values for education=.005). There was no statistically significant difference in the effect sizes between the CIMI group and the control group by age, marital status, working experience, or weekly working hours subgroups (interaction *P* values were .92, .71, .22, and .09, respectively).

## Discussion

### Principal Findings

This study found that, with the exception of anxiety symptoms, most psychological markers significantly improved more in the CIMI group following a 12-week intervention than in the self-guided group. When it came to alleviating sleep disorders, the CIMI was more effective in reducing fatigue and sleepiness. Regarding physiological indications, HRV decreased in both groups but stayed within the range that is considered clinically normal. Overall, the CIMI was found to be effective for improving psychological indicators and sleep symptoms in HCWs, but not in physiological stress indicators.

To the best of our knowledge, despite the fact that numerous stress management interventions have been implemented in workplaces, none of them have been specifically designed for distressed HCWs. As previously stated, developing stress management behaviors would be difficult for HCWs with distress symptoms even if they were aware of the effects of stress and coping strategies. Hence, we developed a CIMI based on knowledge translation theory [22] and SLT [24] to bridge the “knowing-doing” gap in stress management among distressed HCWs. The CIMI specifically created an online context with the goal of changing the HCWs’ negative attitudes and skills through mobile stress management education, a web-based WeChat social network, tailored feedback, and a nurse coach. This study compared the effects of the CIMI and self-guided groups on stress symptoms using physiological, psychological, and sleep disorder indicators.

In terms of physiological indicators of stress, SDNN is a common HRV metric that represents the normal-to-normal SD. SDNN may drop significantly in disorders such as myocardial infarction, indicating collisions with HRV. With a normal SDNN range of 102 to 180 ms, SDNN swings within a specific range under physiological conditions [37,44,45]. Both the intervention and control groups showed isotropic changes in SDNN before and after intervention. There was no statistical difference between the 2 groups. This suggests that the CIMI is primarily concerned with improving subjective markers.

In aspects of psychological stress measures, the CIMI was significantly more effective in improving depression and stress symptoms and elevating the subjective happiness of HCWs. However, we found that the anxiety symptoms in the control group exaggerated significantly from baseline to after the intervention, while there were no significant changes in anxiety symptoms in the intervention group. That means the effect of the CIMI on alleviating anxiety was still unclear. Furthermore, we found that the CIMI helped improve daytime sleepiness and fatigue symptoms significantly. In a word, the CIMI was found to be significantly more effective than the control group in improving both psychological and sleep indicators of stress, as well as parts of psychological stress measures. Although previous research found that human support was more effective than self-guided mobile interventions, the majority of studies did not directly compare self-guided and human support e-therapy [47]. Therefore, this study provided objective evidence of the effect of the CIMI, a novel human support intervention, on alleviating stress symptoms among distressed HCWs.

To explore if our intervention effects varied between participants with different demographic characteristics, we further performed a subgroup analysis. We found that educational level, sex, and occupation may all influence the treatment effects of the CIMI. Participants with postgraduate degrees benefited from the intervention more than those with graduate degrees. This finding was consistent with previous research, which found that highly educated participants were more likely to engage in health-promoting behaviors and use interventions more effectively [48,49]. This could be explained by the fact that those with higher educational levels also tend to have higher socioeconomic status, allowing them to better protect themselves from the risk of poor mental health [50]. Thus, future research should consider how to assist participants with lower educational levels in making better use of interventions. Besides, we found that men benefited more from the interventions than women, which was consistent with prior studies. This may be explained by the fact that women are more likely to assume more family duties, thus they are less likely to have spare time to practice the recommended stress management techniques [51]. Hence, future research should consider how to help female HCWs get support from families so that they can spend enough time on stress management. Interestingly, we discovered that occupation influenced treatment effects, with physicians benefiting from interventions more than nurses. The findings were similar to those of Yildirim et al [52], who identified that physicians exercised more than nurses and had a higher quality of life. Given that nurses face more barriers to adopting health-promoting behaviors and have higher levels of work stress [53], future interventions may need to target this population in order to successfully manage their stress.

Overall, this study found that the CIMI was more effective than self-guided stress management in distressed HCWs. The design’s uniqueness stems from the combination of multiple interventions and target populations. The integration of all elements into a computerized program allowing online crosstalk with participants could be a future CIMI modification [54,55]. It would save labor while also increasing the acceptance of interventions among distressed occupational populations.

### Limitations

This study has several limitations that should be mentioned. First, the controlled, nonrandomized design may bias estimates of treatment effects and cause uncertainty in our findings. As a result, future studies can consider using randomized controlled trials to investigate our findings. Second, because of the COVID-19 pandemic, we failed to balance sex and age during our recruitment process. To avoid any bias caused by demographic characteristics, the Cohen *d* effect was calculated by dividing the between-group adjusted MD by the SDs at baseline in this study. Third, since our study only tested the effectiveness of the CIMI over a 3-month period, it was unclear how long its effects would last. As a result, more research into the long-term effects of human assistance intervention is needed. Last but not least, we recognized that we might not have been able to detect the significant effect of the CIMI on improving HRV measures since we had not been sure that our sample size complied with the requirements for HRV indicators. Therefore, we recommended that future studies examine the influence of CIMI on HRV markers using a larger sample size.

### Conclusions

In this study, we discovered that the CIMI was effective at alleviating stress symptoms in distressed HCWs. This study’s findings may provide objective evidence for the development of an effective mobile stress management intervention for distressed HCWs. Given that this study was a nonrandomized controlled trial that lasted 3 months among distressed HCWs, future research may consider investigating the long-term effects of the CIMI using a randomized controlled trial design.

## Appendix

Multimedia Appendix 1 Printed booklet.

Multimedia Appendix 2 Weekly questionnaire.

## Abbreviations

CIMI: complex interactive multimodal intervention
DASS-21: Depression, Anxiety, and Stress Scale
ESS: Epworth Sleepiness Scale
FAS: Fatigue Assessment Scale
HCW: health care worker
HRV: heart rate variability
JBI: Journal of Biomedical Informatics
MD: mean difference
PSS: Perceived Stress Scale
REDCap: Research Electronic Data Capture
SDNN: SD of all normal-to-normal intervals
SHS: Subjective Happiness Scale
SLT: social learning theory
SMS: stress management strategy

## Data Availability

The data sets generated and analyzed during this study are available from the corresponding author on reasonable request.
