# Supplementary material for: The Effects of a Complex Interactive Multimodal Intervention on Personalized Stress Management Among Health Care Workers in China: Nonrandomized Controlled Study
Source: J Med Internet Res. 2024 Jul 12;26:e45422. doi: 10.2196/45422 (PMC11282381; doi:10.2196/45422)
Supplement: Multimedia Appendix 1 [file jmir_v26i1e45422_app1.pdf]

医护人员压力应对手册

——科学管理工作压力，有效保障职业健康

运动：每天运动 15-20 分钟，一周运动 2 小时

深呼吸训练：每天在不同时间练习深呼吸 5-10 分钟，一分钟 6 个呼吸循环

专注力训练：每天在日常生活如进食、走路、冥想、呼吸中进行专注力训练 15 分钟

第一章

您工作中的慢性压力

1. 压力是什么？

压力是机体应对外界环境刺激时，为维持自身稳态平衡而产生的一种应激反应。过量饮食、饮酒、吸烟或工作超时等都可能成为您日常生活中常见的压力来源。

2. 慢性压力会产生怎样的不良后果？

无论在应对何种类型压力时，机体的自主神经系统功能都发挥着极为重要的作用。压力情境下，个体的交感神经系统（Sympathetic Nervous System, SNS）兴奋性会显著增加，而副交感神经系统（Parasympathetic Nervous System， PNS）的兴奋性则显著降低。之后，被称作为“压力荷尔蒙”的皮质醇也会显著分泌增多。正常情况下，交感神经系统和副交感神经系统功能总能维持在一定的平衡状态（图 1）。

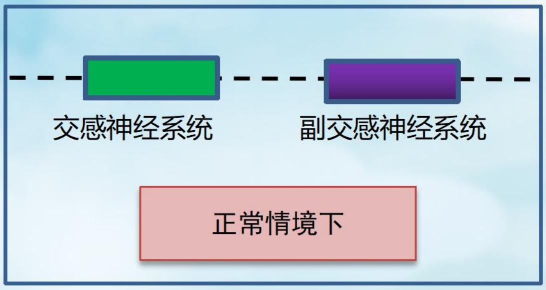

图 1 正常情境中自主神经系统的功能稳态

但慢性持续的压力，如长达数天甚至数周的压力则会对机体产生不良影响（图 2）。在慢性压力情境下，交感神经系统功能和皮质醇分泌浓度均处于较高水平，而副交感神经功能则处于较低水平；长此以往，机体将触发一系列的慢性炎症反应，随着时间

迁移还可显著增加罹患多种慢性疾病的可能性。

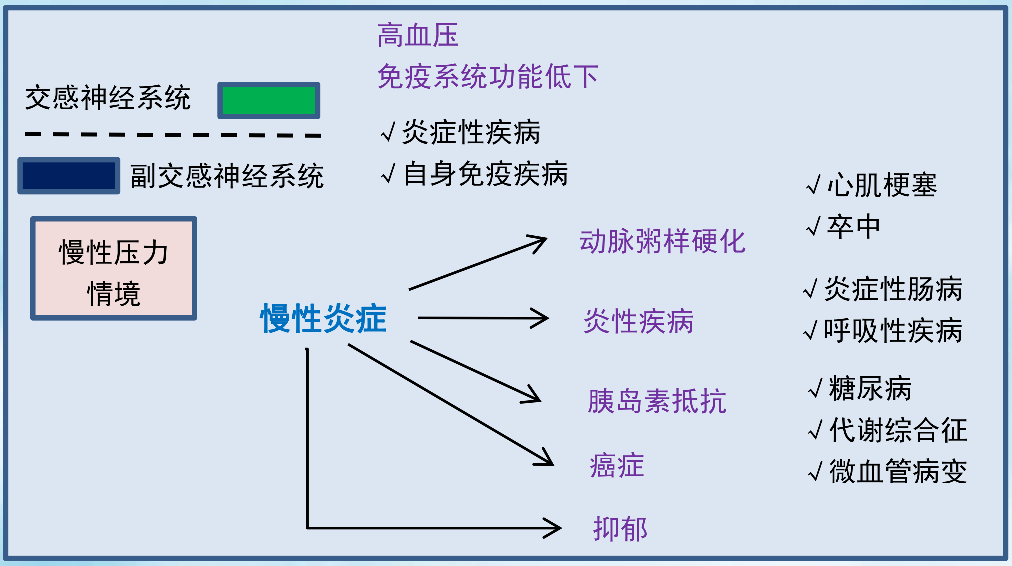

图 2 慢性压力情境对于机体产生的影响

➢ 职业压力不容忽视

职业压力大多可归因于工作任务繁重、情绪压力大、人际关系紧张或团队冲突等，个体常会感受到不堪重负、挫败气馁、焦虑等负性情绪和倦怠感。

对于医护工作者而言，我们应尤其重视夜班这一特殊压力源所带来的不良后果。夜班会严重影响睡眠质量，使个体易产生疲乏感，并诱发慢性炎症反应。尤其是长期处于高强度压力的工作环境下，我们时常容易产生疲惫感、焦虑和抑郁等情绪。

第二章

科学管理职业压力

理论上，化解压力的最佳方法是消除环境中的压力源。例如，保持良好的睡眠和健康饮食，以及改善环境氛围等。但想要完全移除工作场所中的压力源并不可行。因此，对于职业人群而言，如何运用最为简便的方法，科学合理地管控压力，显得尤为重要。

在本项目中，我们向您推荐以下三种有效缓解压力的途径：

（1）规律运动；

（2）深呼吸练习；

（3）专注力训练。

根据我们的指引，您可以轻松掌握各项缓解压力的方法；您也根据自己喜好，对上述提及的方法进行创造性地组合运用。例如在您进行体育锻炼（如散步时）时，您也可以练习深呼吸和（或）专注力以提升自己对于周围环境的感知能力，从而达到更佳的减压效果。

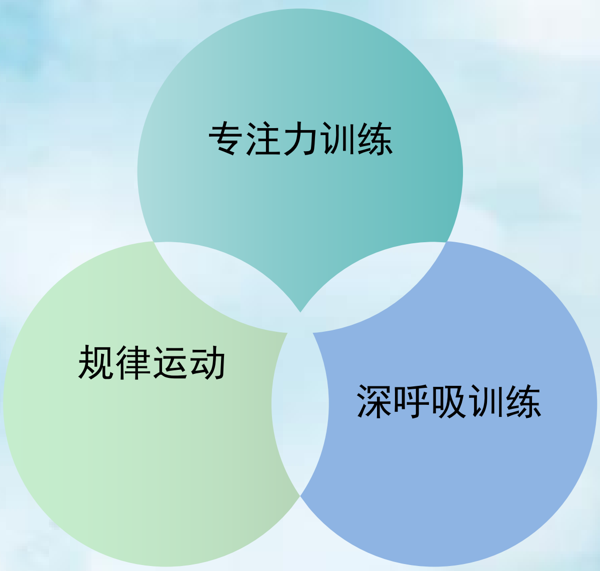

图 3 缓解压力的有效途径

第一节 缓解压力之规律运动篇

一、选择规律运动的理由

1. 规律运动已被证实是缓解压力的最为有效方法之一；

2. 规律运动可显著减轻机体慢性炎症反应；

3. 规律运动可改善糖尿病、抑郁、认知障碍和高血压等疾病；

二、应该选择哪种规律运动方式呢？

1. 个体化选择：没有哪种体育运动适合所有人，换言之，您可以根据自身情况，选择易于长期坚持的运动方式；

2. 融入生活：让运动成为日常生活的必需品，逐步建立规律性的运动习惯，实现生活方式的转变；

3. 规律运动：每天 15-20 分钟的步行锻炼效果要胜过每周 1 次 2 小时的剧烈运动；

4. 合理锻炼：运动并不必让人感到精疲力竭，若锻炼方式不当，运动本身还会成为额外的压力源；

5. 找到适合自己的运动：推荐您选择适合自己的最佳运动方式，可以是打球、慢跑、散步，甚至是在遛狗时小跑一会儿；

6. 乐在其中：好的运动可以给您带来身体心理的双重愉悦感，并会让您感受到内心的平和；

7. 舒缓放松：运动是您减压放松的途径，在运动时您也可以尝试将深呼吸和专注力练习融入其中；

8. 数据监控，保质保量：在运动时，您可以运用手环监测自己

的心率变化，以保障运动效果与心脏健康；当我们的心率维持在60%~70%的最大心率（最大心率=220-年龄），并持续运动30分钟~60分钟，将最有利于热量的消耗和心肺功能的提升。

以下我们列出来几条您在运动中需要注意的事项：

①注意舒展四肢及躯干，

②进行下蹲、旋转及跳跃运动等动作时，注意保持身体的灵活性和平衡性，

③我们推荐您参与健美操、太极拳等有氧运动，

④您也可以选择团体类体育运动，如跳广场舞等。

第二节 缓解压力之深呼吸训练篇

一、选择深呼吸训练的理由

科学研究证据表明深呼吸能有效减缓心率和降低焦虑感。

二、 怎样训练深呼吸技巧？

深呼吸技术掌握起来是相当容易的，但若在练习过程中，您过于关注呼吸的频率，而忽视了自己的呼吸节奏，呼吸练习则会让您感到有些不适；

1. 放轻松，根据您自己的呼吸节奏来调节呼吸频率是深呼吸练习的关键所在；

2. 下面的几个小窍门将会帮助您更容易地掌握深呼吸技巧：

（**1**）找一个方便练习深呼吸的地方，选择您喜欢的姿势，您可以选择坐在、站着或是在走路时练习；

（**2**）在开始练习时您可以先尝试增加吸气的幅度，每次都尝试更深的吸气，并注意跟随自己的呼吸节律；

（**3**）再尝试增加自己的呼气幅度，同样也要注意和自己的呼吸节律合拍；

（**4**）请牢记，保持舒适并跟从自己的呼吸节律进行练习；

（**5**）经过几轮深呼吸练习，您应该能达到每分钟 **6** 次的深呼吸频率（每个呼吸周期持续 **10** 秒左右）；

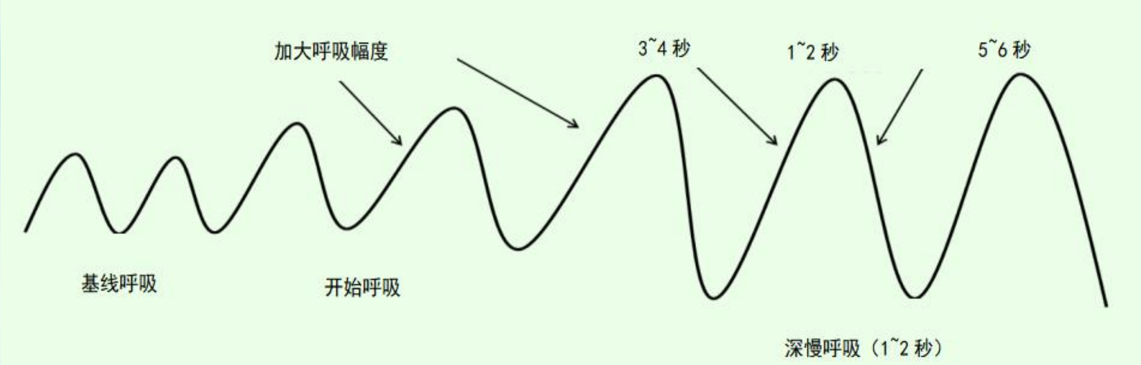

图 4 深呼吸训练

3. 若您在练习时感到有点头晕，你可以暂停一下，并试着降低自己的呼吸幅度，等到感觉舒服一些后再继续练习；经过多次练习与尝试，您的身体感觉将会帮您找到最适合自己的深呼吸训练方式；

4. 定时练习：坚持每天 **3** 次，每次 **5** 分钟的练习，您可以随时随地

练习深呼吸技巧，只要自己觉得方便就好。

第三节　缓解压力之专注力训练篇

在许多冥想练习中，例如近 10 年来颇为流行的正念冥想中，我们都可以看到专注力训练的身影，专注力训练是一种通过提高自身感知和觉察能力的有效减压方式。

一、选择专注力训练的理由

1. 专注力训练可帮助您更好地觉察到身体的细微感受：

（1）专注力训练有助于提高您在工作、家庭、社会生活中的觉察力，正因如此，许多运动员也正在通过专注力训练来提升自己的体育战绩；

（2）在不利的情境中，更好的觉察力将有助于您更好地控制自己的情绪，因而专注力训练也被诸多冠军视为自己克服逆境的秘密武器。

2. 外周环境（如声音、工作压力、紧张氛围等）和我们的身体、情绪和灵性健康息息相关，专注力训练能增强您对于外界环境的感知能力，并有助于您避免减少的应对方式；

3. 专注力训练能让您逐渐获得免受外界环境和自身情绪干扰的能力，让您拥有更强大的内在力量去直面逆境。

二、关于专注力训练的一些建议

1. 您可以将专注力训练带入到每一件日常活动之中，如进食、喝茶、散步等活动中；您也可以尝试将专注力带入到多种感知体验中，例如在仰望天空、聆听音乐、细闻米饭清香、舒缓按摩时练习专注力；您甚至可以通过发挥自己的想象来练习专注力；

2.专注力训练的关键在于将注意力聚焦于某种感官体验上，比如，在散步时，您用心感受步行过程中自己身体每个部位的活动，例如足部接触地面的感觉，双腿支撑的力量，上臂和躯干各个部位的协调活动等；亦或，在吃饭时，您可悉心捕捉米饭散发的香味，体验每粒米饭的触感和材质，并认真品尝不同食材带来的味觉体验，在咀嚼和吞咽过程中，您甚至还能感觉到各种食材的各种形态变化——关注这些您或曾忽视的细微感受，正是专注力训练的范畴。

3. 您也可以通过自己的兴趣爱好来尝试专注力的训练，如在绘画，书法，练习乐器和听音乐中提升自己的专注力。

三、专注力训练的长期影响

1. 长期坚持专注力训练能让您成为自己意识的主宰者；

2. 专注力训练有助于缓解压力、焦虑等负性情绪，提升您对外部世界的美好感知，有助于建立积极地工作和生活态度，以及良好的人际关系。

第四节　小结

1. 职业压力不容忽视，它是压力和负性情绪的重要来源，因此会对您的身体造成潜在不良影响；

2. 改善工作环境固然有助于缓解职业压力，但考虑到患者对于医护服务的需求是 24 小时全年无休的，以及医院环境中存在诸多不利因素均会对医护人员的情感心灵健康造成不利影响，医院环境将会作为一种特殊的压力源持续存在且难以改变；

3. 因而，从个体层面出发找寻最适合自己的压力控制方式显得尤为重要，否则压力将会影响到我们生活的方方面面，甚至波及我们的家人、朋友和同事；

4. 本手册中推荐的 3 种简单易行的压力，有的方法，每天仅需几分钟即可完成，您可以在工作间隙实践；随着您的练习次数和时间的增加，它们将成为您生活的不可或缺的一部分，促进长期生活习惯的转变和建立；

5. 您可以灵活运动本书中介绍的技法，单用或综合运用以上 3 种方法，没有最好的选择，只有最适合您的选择；

7. 最后，我们衷心希望，我们可以帮助您找到最适合自己的压力管理方式。当下，您将成为自己压力管理和健康生活的主宰者，而我们很荣幸成为您学习压力管理征程的领航员！让我们一起携手共进，科学管理工作压力，有效保障职业健康！
