# Supplementary material for: The Effects of a Complex Interactive Multimodal Intervention on Personalized Stress Management Among Health Care Workers in China: Nonrandomized Controlled Study
Source: J Med Internet Res. 2024 Jul 12;26:e45422. doi: 10.2196/45422 (PMC11282381; doi:10.2196/45422)
Supplement: Multimedia Appendix 2 [file jmir_v26i1e45422_app2.docx]

**WEEKLY DIARY**

**ID No:** |__|__|__| **Site No**: |__| **Date:** |_|_| |_|_| |_|_|_|_| DD. MM YYYY

1. **THIS PAST WEEK (last 7 days):**
2. **How often have you worked at night during this past week?** |__| (from 0 to 5)
   1. When was your last working night?

Last night |_|, Night before |_|, 2 nights ago |_|, >2 nights ago |_|

1. **How has this past week been regarding stress at work?**

Not at all stressful Extremely stressful

1

2

3

4

5

6

7

8

9

10

0

1. **During this past week, have you experienced unexpected stressful events in your life (could be related to personal matters, family, anything)?** Yes |_| No|_|

If Yes: Do you think this event will affect your life and may be a source of stress for the next few weeks? Yes |_| No|_| May be |_|

Can you describe: _________________

**NOTE:** If the event is important, it may require a specific description using the “Unexpected Event Description Form”

1. **REGARDING THE PRACTICE OF STRESS-MANAGEMENT EXERCISES**
2. **How was this past week regarding the practice of Physical activity exercise?**

Very easy very difficult

1

2

3

4

5

6

7

8

9

10

0

**What do you find difficult? ____________**

**How often have you done some physical activity exercise this past week?**

Never |_| |1 | 2 | 3 | 4 | 5 | 6 | 7 | times per week >7 times/week |_|

**Do you find Breathing exercise useful for you?** Yes |__|; No |__|; Not sure |__|

**If Yes, do you find it:** Very useful |__|; fairly |__|; a little |__|; not much |__|

1. **How was this past week regarding the practice of Deep breathing exercise?**

Very easy very difficult

1

2

3

4

5

6

7

8

9

10

0

**What do you find difficult? ____________**

**How often have you done some deep breathing exercise this past week?**

Never |_| |1 | 2 | 3 | 4 | 5 | 6 | 7 | times per week >7 times/week |_|

**Do you find Breathing exercise useful for you?** Yes |__|; No |__|; Not sure |__|

**If Yes, do you find it:** Very useful |__|; fairly |__|; a little |__|; not much |__|

1. **How was this past week regarding the practice of Body mindfulness exercise?**

Very easy very difficult

1

2

3

4

5

6

7

8

9

10

0

**What do you find difficult**? ____________

**How often have you done some body mindfulness exercise this past week?**

Never |_| |1 | 2 | 3 | 4 | 5 | 6 | 7 | times per week >7 times/week |_|

**Do you find Mindfulness exercise useful for you?** Yes |__|; No |__|; Not sure |__|

**If Yes, is it:** Very useful |__|; fairly |__|; a little |__|; not much |__|

***Would you like to discuss with the coach your experience with stress management exercises?*** Yes |_| No |_|

1. **REGARDING FEELINGS AND EMOTIONS DURING THE LAST WEEK:**

**During the last week, could you score your perception, regarding the following symptoms you perceive** = from “0” = “not at all”, to “10” = “extremely”:

1. **Feeling tired (in general)?**

Not at all Extremely

1

2

3

4

5

6

7

8

9

10

0

1. **Feeling happy (in general)?**

Not at all Extremely

1

2

3

4

5

6

7

8

9

10

0

1. **Feeling anxious (in general)?**

Not at all Extremely

1

2

3

4

5

6

7

8

9

10

0

1. **Feeling sad, depressed (in general)?**

Not at all Extremely

1

2

3

4

5

6

7

8

9

10

0

1. **Feeling tense: How do you score your body tension today?**

No tension Extreme tension

1

2

3

4

5

6

7

8

9

10

0

1. **REGARDING SLEEP QUALITY DURING LAST WEEK:**
2. **Overall, how would you rate your sleep quality in the last week?**

Very Good |__|; Fairly Good |__|; Fairly Bad |__|; Very Bad |__|

1. **In general, have you experienced difficulties to fall asleep at night?**

Never |__|; Rarely |__|; Sometimes |__|; Always |__|

1. **In general, have you experienced difficulties to get up in the morning?**

Never |__|; Rarely |__|; Sometimes |__|; Always |__|

1. **In general, have you experienced waking up in the middle of the nigh with difficulty to sleep again?**

Never |__|; Rarely |__|; Sometimes |__|; Always |__|

1. **On average, could you estimate the number of sleep hours per night?**

<5 hour/night |__|; 6h/n |__|; 7h/n |__|; 8h/n |__|; > 8h/n |__|;

1. **Overall, this past week, do you think you had enough sleep (quantity)?**

Yes, I had enough sleep |__|; Missing sleep a lot |__|; Missing a bit |__|

1. **This past week, because of lack of sleep, did you experience dozing in different situations?** (check all that apply) Reading a book |__|; Watching TV |__|; Talking to people |__|; on a car or the bus |__|; Other circumstances |__|
2. **This past week, did you have to take some sleeping pills to be able to sleep?**

Yes |_| No|_|

1. **Have you used other ways to relax (like drinking some wine or smoking) to be able to sleep?** Yes |_| No |_|
